# Comparison of Carotid Ultrasound Indices and the Triglyceride Glucose Index in Hypertensive and Normotensive Community-Dwelling Individuals: A Case Control Study for Evaluating Atherosclerosis

**DOI:** 10.3390/medicina54050071

**Published:** 2018-10-11

**Authors:** Javad Alizargar, Chyi-Huey Bai

**Affiliations:** 1 School of Public Health, College of Public Health, Taipei Medical University, Taipei 11031, Taiwan; 2 Department of Public Health, College of Medicine, Taipei Medical University, Taipei 11031, Taiwan

**Keywords:** carotid intima media thickness, hypertension, atherosclerosis, carotid artery, risk factor, insulin resistance, triglyceride glucose index

## Abstract

*Background and objectives*: Hypertension (HTN) is an important risk factor for cardiovascular diseases. High blood pressure is a major cause of atherosclerosis which leads to myocardial infarction and stroke. Insulin resistance (IR) is correlated with HTN and atherosclerosis. To determine differences between the effects of HTN on the intima media thicknesses (IMTs) of the internal (ICA), external (ECA), and common carotid arteries (CCA), and evaluate the carotid plaque presence between hypertensive and normotensive individuals, a case-control study was designed among community-dwelling individuals. The relationship between the triglyceride glucose (TyG) index and atherosclerosis was also investigated in this study. *Materials and Methods*: Data from 77 hypertensive and 199 normotensive individuals were analyzed in this study. *Results*: The IMTs of the CCA, ICA, and ECA, and the TyG index were all higher in hypertensive individuals compared to the control group (all *p* < 0.05). After controlling for age, sex, the body-mass index, and TyG index, HTN was an independent predictor of a high CCA IMT (odds ratio (OR) = 2.48; 95% confidence interval (CI) = 1.24–4.93) and presence of plaque (OR = 2.36; CI = 1.15–4.85) in the carotid artery. *Conclusions*: HTN was an independent risk of carotid IMT thickening and atherosclerosis. TyG index could only predict the CCA IMT independent of other risk factors (OR = 2.09; CI = 1.07–4.09).

## 1. Introduction

Atherosclerosis is a chronic condition that can cause death due to vascular disease. It causes an accumulation of fatty streaks in arterial walls, which develop into atheromas and plaque. Depending on the site, after rupturing, this plaque may cause occlusion. Ischemic heart disease, stroke, and peripheral arterial disease are some example consequences. These conditions impose great burdens on the health of community-dwelling individuals [1]. Different risk factors have been reported for atherosclerosis. Among all the risk factors, hypertension (HTN) can affect atherosclerosis on different levels [2]. HTN can cause endothelial dysfunction, fatty streaks accumulation, subclinical atherosclerosis, atherosclerosis progression, and the ultimate rupture of plaque [3]. It is thought to be a main factor in the hypertrophy of the media layer in the carotid artery, which can be detected by ultrasonography (US) [4].

Evaluation of HTN in atherosclerosis can be considered an important issue in cardiovascular disease risk prediction. Many methods have been proposed for atherosclerosis evaluation. The intima media thickness (IMT) of the carotid artery is measured by US and has been used as a marker of subclinical atherosclerosis for years [5]. As different parts of the carotid artery can be used to determine the thickness, some studies suggested that the common carotid artery (CCA) provides more reliable and reproducible measurements. However, changes in the CCA due to HTN can differ from those of the internal carotid artery (ICA) and external carotid artery (ECA). Different ethnicities may have different implications of thickening in different parts of the carotid artery due to HTN [6]. The literature is ambiguous as to the relationship of HTN with the presence of carotid plaque, as some studies showed a significant association between HTN and plaque presence [7], while other studies found no association [8]. As carotid thickening occurs in the early stages of atherosclerosis and plaque forms at later stages, it is said that the carotid IMT is a marker of early-stage subclinical atherosclerosis, and plaque presence is a marker of late-stage subclinical atherosclerosis [9].

Aside from HTN, insulin resistance (IR) is another known mechanism underlying atherosclerosis, even in the absence of diabetes mellitus (DM) and hyperglycemia [10] as in atherogenesis and plaque formation. The triglyceride glucose (TyG) index is calculated using fasting glyceride and glucose levels and is a good marker of IR [11]. The relationship between the TyG index and atherosclerosis was proven in postmenopausal women [12] and needs further assessment in community-dwelling individuals. The TyG index’s relationship with HTN was the focus of a recent investigation [13]. A large cohort study proposed the TyG index as an independent factor in the development of HTN in normotensive individuals [13,14].

As there is no consistency in the relationship between HTN and the presence of carotid plaque, and to determine differences in the effects of HTN on the IMTs of the ICA, ECA, and CCA, a case-control study was designed among community-dwelling individuals in Taiwan. We also investigated the association of IR, measured by TyG index with atherosclerosis as our secondary objective in this study.

## 2. Experimental Section

This study used data of a prospective cohort study conducted on community-dwelling individuals and aimed to evaluate the cardiovascular and cerebrovascular risk factors. The study was conducted in accordance with the Declaration of Helsinki, and the protocol was approved by the Ethics Committee of Taipei Medical University with Institute Review Board (IRB) (reference numbers 94E-183, 94E-198, and 96E-004). The method of data collection and the exclusion criteria were explained elsewhere [15]. In summary, starting in 2005 and 2006, residents of twelve towns near Taipei (six towns in Shihlin District and six towns in Wenshan District) were invited to participate in the study. After excluding individuals who were <30 years old, had incomplete questionnaires, had a prior history of cancer, chronic kidney disease, stroke or myocardial infarction, smoking or refused to allow blood to be drawn or to have a carotid US, 543 individuals were sent to have a carotid US. The Carotid US was performed by the same cardiologist in all individuals. Among the 543 individuals, 312 had the following criteria: Carotid US and laboratory tests were done on the same visit, data regarding their history of HTN and hypertensive medication were accessible, and they did not have a drug history of angiotensin II receptor blockers, calcium channel blockers, and Statin drugs. Individuals who had confirmed DM by a physician or were using DM medication were excluded (*n* = 36). Our final analyzed dataset consisted of 77 hypertensive and 199 normotensive individuals. Written consent was obtained from all participants in the original study, no name was published in any form, all participants’ data remained confidential after data collection, and each participant was recognized by a code.

To ensure that the sample size was sufficient for our study, according to the study of Naseh et al. [16], we calculated the minimum sample size for comparing two means as a minimum of 65 in the case and 130 in the control group (95% confidence interval, 80% power, and a ratio of control to case = 2) based on the following formula:

*n* = *(Z_α_*_/2_ + *Z_β_*)^2^*2*σ^2^/*d*^2^, where *Z_α_*_/2_ is the critical value of the Normal distribution at *α*/2, *Z_β_* is the critical value of the Normal distribution at *β*, *σ*^2^ is the population variance, and *d* is the difference between carotid IMT measurement in cases and controls.

Data, including the age, sex, body-mass index (BMI), systolic blood pressure (SBP), and diastolic blood pressure (DBP), were extracted from the charts. An individual was categorized into the hypertensive case group if he or she had confirmed HTN or used hypertensive medication. Other individuals were placed in the control group.

Certain laboratory results from the blood test were also included in this study. These tests were as follows: Fasting blood sugar (FBS, mg/dL), uric acid (URCA, mg/dL), blood urea nitrogen (BUN, mg/dL), creatinine (Cr, mg/dL), triglyceride (TGL, mg/dL), cholesterol (CHOL, mg/dL), low-density lipoprotein (LDL, mg/dL), high-density lipoprotein (HDL, mg/dL), C-reactive protein (CRP, mg/L), and glycated hemoglobin (HBA1c; %). Standard methods (using an X-1500-Sysmex, Deckman AU5800, and Tosho HLC-723G8 automated glycol-hemoglobin analyzer) were used to analyze these parameters. The TyG index was calculated as ln(TGL × FBS/2).

B mode carotid US using a SONO 5500 instrument (HP, Palo Alto, CA, USA) was conducted on each participant. The flow (mL), end diastolic velocity (EDV; cm/s), and peak systolic velocity (PSV; cm/s) were measured in one cardiac cycle. The mean velocity (MV) was calculated as (PSV + 2 × EDV)/3, and the resistance index (RI) as (PSV − MV)/PSV. The cervical portion of the CCA, the ICA beyond the carotid bulb, and the ECA were used to measure the IMT on both sides, and numbers of plaque in the carotid arteries were measured by a cardiologist. The total amount of carotid plaque was calculated by adding the numbers of plaque on the right and left sides in each person and is presented as the carotid plaque score (cPS).

To dichotomize the value of the carotid IMT into high and low, the 75% quartile was used. Numbers and percentages were reported for categorical values, and the mean and standard deviation (SD) were used for continuous ones. Fisher’s exact test and an analysis of variance (ANOVA) were used to analyze differences between the case and control groups. A nonparametric test (Mann-Whitney U test) was used when the assumption of normality was not met. Data are expressed as mean and Standard Deviation (SD) if they are normally distributed (Shapiro-Wilk test *p* value > 0.05) or median and interquartile range (IQR) if they are not normally distributed (Shapiro-Wilk test *p* value < 0.05). Separate logistic regressions were used to indicate the IMTs of the CCA, ICA, ECA, and the presence of carotid plaque as dependent variables, after controlling for age, sex, HTN history, and the TyG index. To evaluate the correlation between the TyG index and atherosclerosis, a Pearson correlation test was used. Receiver operating characteristic (ROC) curves and odds ratio (OR) plots of the CCA IMT and plaque presence are shown using a stepwise logistic regression. Independent variables were the same as in the previous logistic regressions model. A *p* value of <0.05 is considered significant, and SAS vers. 9.4 (SAS, Cary, NC, USA) was used for all analyses. The power of the study was calculated with the method of normal approximation for comparing two means and an unmatched case-control using the free online Openepi platform (http://www.openepi.com/Menu/OE_Menu.htm).

## 3. Results

Data of 276 individuals were analyzed in this study: 77 in the case group with a history of HTN, and 199 without a history of HTN. Males comprised 45.4% of the case group and 60.8% of the control group. Results of demographic characteristics and carotid US are shown in Table 1.

For evaluation of the correlation between IR and atherosclerosis, the Pearson correlation was calculated between TyG and cPS and IMT in different areas of the carotid artery, as shown in Table 2.

Results of the logistic regression analysis for detecting high (>75% quartile) CCA, ICA, and ECA IMTs, and also the plaque presence are shown in Table 3. Males had significantly higher odds of having high CCA, ICA, and ECA IMTs (OR = 3.5; CI = 1.8–6.8; OR = 5.3; CI = 2.9–9.7; and OR = 3.2; CI = 1.7–6, respectively) but not plaque presence (OR = 1.8; CI = 0.9–3.3). HTN significantly increased the odds of a high CCA IMT (OR = 2.4; CI = 1.2–4.9), and also increased the odds of having more than two-fold higher carotid plaque (OR = 2.3; CI = 1.1–4.8). The TyG index was only the independent predictor of a high CCA IMT (OR = 2; CI = 1.2–4.9).

**Figure 1:**
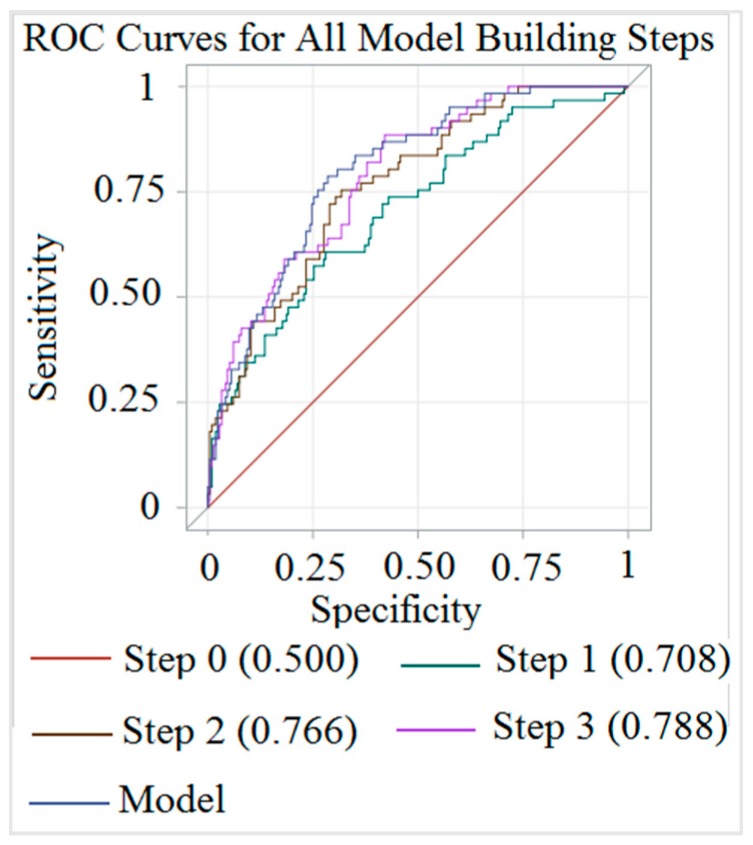
Receiver operating characteristic (ROC) diagram of stepwise multiple logistic regressions for high common carotid artery intima media thickness (CCA IMT) in community-dwelling individuals. (Step 1: Adding age, step 2: Adding sex, step 3: Adding the triglyceride glucose (TyG) index, and model: Adding hypertension (HTN) to step 3).

**Figure 2:**
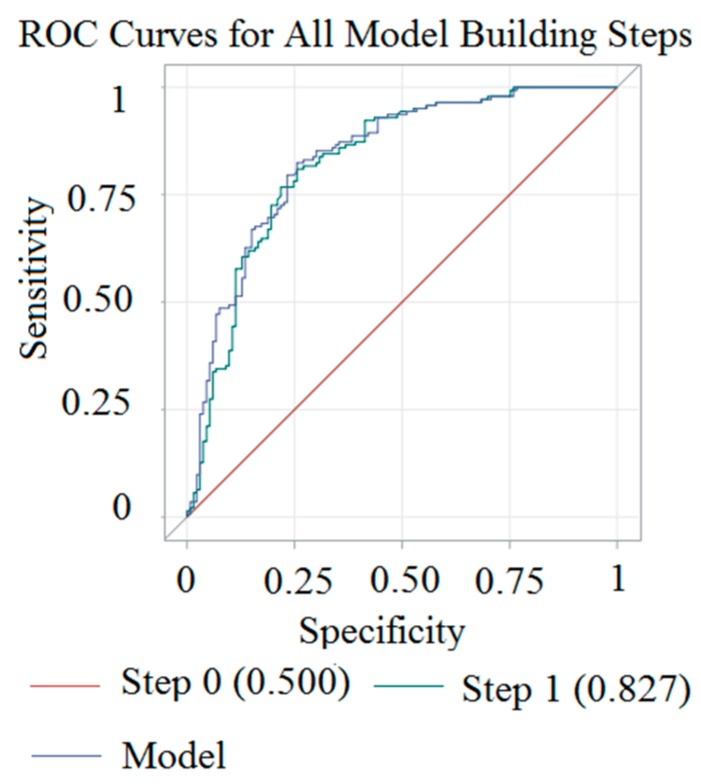
Receiver operating characteristic (ROC) diagram of stepwise multiple logistic regressions for carotid artery plaque presence in community-dwelling individuals (Step 1: Adding age, and model: Adding hypertension (HTN) to step 1).

The stepwise logistic regression for a high CCA IMT showed an area under the curve (AUC) of 80.1% using age, sex, the TyG index, and HTN history. The stepwise logistic regression for plaque presence showed an AUC of 83.7% using age and HTN history. Detailed results are given in Figure 1 and Figure 2.

The power of the study for the IMTs of the CCA, ECA, and ICA, and for a high cPS and plaque presence in a two-sided test with a 95% confidence interval (CI), was calculated to be 100%. For the TyG index, the power was 83.22%.

## 4. Discussion

This case control study on 77 hypertensive and 199 control individuals showed that the flow of B mode US in hypertensive individuals was significantly lower than that of the control group. IMTs of the CCA, ICA, and ECA, and the TyG index were higher in hypertensive individuals compared to the control group. There was a greater plaque presence in hypertensive individuals that in the non-hypertensive control group. The TyG index was significantly correlated with the cPS, and IMTs of the CCA, ICA, and ECA. After controlling for age, sex, and the TyG index, HTN was an independent predictor of a high CCA IMT and plaque presence in the carotid artery. Age, sex, the TyG index, and an HTN history could predict the CCA IMT with 80.1% accuracy, and age and an HTN history could predict the presence of plaque with 83.7% accuracy.

In a study of 43 hypertensive and 43 normotensive individuals, Naseh et al. [16] found that all parts of the carotid artery in the case group had higher IMTs than the controls. Our study results are in accordance with their study. By increasing the pressure of blood inside the arterial lumen, large arteries go through a process of remodeling. This increases the size of smooth muscles and also causes proteins like collagen and fibronectin to accumulate in the extracellular matrix. This results in stiffened arteries that increase cardiovascular events [17]. Particularly the carotid IMT and to a greater extent, the presence of carotid plaque are reported to be risk factors for cardiovascular events, independent of other risk factors [18]. Our study showed that HTN is an independent risk for an excessive CCA IMT with an OR of 2.4. Mackinnon et al. [19] showed that HTN is a risk factor for a greater CCA IMT in both blacks and whites. However, HTN lost its association after controlling for type-2 DM, the lipid profile, and other cardiovascular risk factors in their study.

In our study, the plaque presence was higher in the HTN group compared to the controls. These results are in accordance with other studies [20]. Our study showed that HTN increases the odds of the presence of carotid plaque by 130.6%. We also observed that the hypertensive case group in our study had a higher CCA IMT compared to the control group. Plaque presence is considered an important determinant of atherosclerosis detection in community-dwelling individuals. These results prove the “response to injury” hypothesis, which states that HTN disrupts the normal overlying endothelium with the involvement of chemical agents. This injury can aggregate oxidized lipids, platelets, and smooth muscles, and be a basis for plaque formation [21].

Our study, in accordance with other studies [6,16], showed that HTN increases the CCA, ICA, and ECA IMTs. However, only in the CCA was this increase independent of other risk factors. As HTN is the main risk factor for atherosclerosis, it can be concluded from these results that the CCA IMT is a better indicator of atherosclerosis, as its relationship with HTN was independent of other risk factors.

The presence of carotid plaque is not the same in every part of the carotid artery. The ICA and bifurcating portion of the carotid artery are more atherogenic [22]. The plaque presence of different parts of the carotid artery was not evaluated in detail in our study and can be considered one of the limitations of this study. Another limitation is that the plaque area that would be a better indicator of atherosclerosis was not measured in our study.

It was found that being of male gender was an independent risk factor for high IMTs in the CCA, ICA, and ECA but not for the presence of carotid plaque. These results are in accordance with other studies [19,20,22]. Our study showed that age was independently associated with a high CCA IMT and the presence of carotid plaque. Although significant, ORs in our study (1.06 for the CCA IMT and 1.14 for plaque presence) were rather small compared to those of other studies [22]. This might have been because we considered age to be a continuous variable and did not categorize it into different subgroups.

Our study showed that the TyG index was significantly higher in HTN cases. Li et al. [13], in a 2017 study of 2680 normotensive community-dwelling individuals with four years of follow-up showed that the TyG index can be an independent risk factor for developing HTN. Our study is in agreement with that study but ours was a cross-sectional analysis and had no follow-up information. They also controlled for salt intake, which is a very important factor in HTN. We did not control for underlying factors, and that is another limitation of our study. We found that the TyG index can be an independent predictor of the CCA IMT, which is a marker of early-stage subclinical atherosclerosis. We found that the TyG index was not a predictor of late-stage subclinical atherosclerosis as it reflects the presence of plaque. The relationship between the TyG index and atherosclerosis has, so far, not been studied in detail in the literature. It might be linked to IR, and the TyG index is a marker of IR. Although there are other markers of IR, such as the homeostasis model assessment of insulin resistance (HOMA-IR), the HOMA-IR is an IR index in the liver and TyG is an IR index of muscles. Therefore, using the TyG index as a predictor of carotid atherosclerosis can be explained by this fact [23,24]. Lambrinoudaki et al. [12], in a study of 473 non-diabetic postmenopausal women showed that TyG was associated with the carotid IMT with a Pearson correlation of 0.15. They used a combined IMT (CCA, carotid bulb, and ICA average on left and right sides) as an index for atherosclerosis. Our study showed a higher correlation in all three measured segments (CCA, ICA, and ECA), and showed that the TyG index was also significantly associated with cPS, see Table 2. The relationship between the TyG index and CCA IMT was more robust in our study, where it was found to be independent of age, sex, the BMI, and HTN history, see Table 3. When controlling for these underlying factors, relationships of the ICA, ECA, and plaque presence with the TyG index disappeared. So we can say that TyG can only predict early-stage subclinical atherosclerosis as independently measured by the CCA IMT.

The effects of diet on cardiovascular risk have been discussed in the previous studies [25,26,27]. Maiorino et al. [26], in a randomized trial, showed that special diets can prevent the progression of subclinical atherosclerosis. Another study showed that diet quality does not have prospective relevance for IMT in younger adulthood [27]. Therefore, more studies are needed to elucidate this relationship in community individuals. Another important risk factor for cardiovascular disease is smoking [28]. Smoking has a negative effect on atherosclerosis by increasing inflammation, thrombosis, and oxidative stress [29]. So studies to evaluate the effects of smoking on IMT and cPS are needed to emphasize the importance of this risk factor.

One of the limitations of our study was that we could not analyze the plaque composition in our study subjects. As plaque composition can be a better indicator of cardiovascular outcome [30], further studies that include plaque composition in the evaluation of atherosclerosis are recommended. Apart from the limitations already mentioned, this study has certain strengths and additional limitations. This is the first study to evaluate the effects of an HTN history as a risk factor for the thickening of different parts of the carotid artery and the presence of carotid plaque. We additionally evaluated the TyG index’s relationships with subclinical atherosclerosis indexes and also compared it to the hypertensive case and to normotensive control groups. This study could not provide a causal relationship. For this, a cohort study is needed. Despite a moderate sample size, the power was more than 80% for our study objectives. As we had excluded smokers in this study and did not collect data regarding the diet of the participants, the effects of smoking and diet could not be assessed in this study. Studies that consider these two factors are suggested. A study of the progression of the carotid IMT over several years between the hypertensive and control groups is recommended. Such a study would further prove the association of HTN with atherosclerosis in different parts of the carotid artery and further clarify the underlying causes of atherosclerosis. Careful consideration of IR mechanisms would also help clarify the exact mechanisms by which the TyG index contributes to atherosclerosis and HTN.

## 5. Conclusions

The mean flow of B mode carotid US in the hypertensive case group of our study was higher than that of the control group, and the case group had higher IMTs in the ICA, ECA, and CCA compared to the control group. The effect of HTN on the IMT was independent of other risk factors in the CCA but not in the ICA or ECA. HTN, being the main determinant of atherosclerosis, makes the CCA IMT more reliable than other parts of carotid artery in assessing atherosclerosis. HTN also increases the odds of plaque presence in healthy community-dwelling individuals independent of other risk factors. This can be explained by the “response to injury” hypothesis of plaque formation in the carotid artery. IR evaluated by the TyG index was higher in non-diabetic healthy community-dwelling individuals in the hypertensive group compared to the control group without a history of HTN. The TyG index was significantly associated with the carotid IMT but could only predict early-stage subclinical atherosclerosis independent of the HTN history, age, sex, and BMI.

## Figures and Tables

**Table 1:** Characteristics of community-dwelling individuals in the case (with hypertension) and control (without hypertension) groups.

Variable	Total	Case (*N* = 77)	Control (*N* = 199)	*p*
**Men, *n* (%)**	156 (56.52)	35 (45.4)	121 (60.8)	0.02
**Age, mean ± SD, years**	56.15 ± 10.65	62.5 ± 8.26	53.68 ± 10.47	<0.01
**BMI, mean ± SD, kg/cm^2^**	23.39 ± 3.04	24.52 ± 2.75	22.95 ± 3.04	<0.01
**Flow, mean ± SD, mL**	220.33 ± 35.89	212.9 ± 33.23	223.22 ± 36.55	0.03
**RI, mean ± SD**	0.65 ± 0.42	0.65 ± 0.04	0.65 ± 0.04	0.41
**ECA Diameter, mean ± SD, cm**	0.35 ± 0.03	0.37 ± 0.03	0.35 ± 0.03	<0.01
**ICA Diameter, median (IQR), cm**	0.42 (0.41–0.45)	0.44 (0.41–0.45)	0.42 (0.40–0.44)	<0.01
**CCA Diameter, median (IQR), cm**	0.56 (0.53–0.60)	0.60 (0.56–0.64)	0.55 (0.52–0.59)	<0.01
**CP presence, n (%)**	142 (51.45)	60 (77.92)	82 (41.21)	<0.01
**High cPS, n (%)**	58 (21.01)	34 (44.16)	24 (12.06)	<0.01
**CRP, median (IQR), mg/L**	0.07 (0.03–0.15)	0.09 (0.05–0.18)	0.07 (0.03–0.14)	0.006
**TyG index, mean ± SD**	8.38 ± 0.56	8.52 ± 0.48	8.32 ± 0.58	<0.01
**SBP, median (IQR), mmHg**	119.25 (109.50–133.00)	140 (130.5–149.5)	114.5 (106–125)	<0.01
**DBP, median (IQR), mmHg**	77.5 (69.5–84.75)	86.5 (80.5–90.5)	74.5 (67.5–80.5)	<0.01
**Pulse, median (IQR), bpm**	68.25 (63–75)	66 (60–71)	69 (63.5–75.5)	0.012
**FBS, median (IQR), mg/dL**	89 (83.5–95)	93 (88–99)	88 (82–94)	<0.01
**URCA, median (IQR), mg/dL**	5.4 (4.5–6.5)	6 (5.2–6.7)	5.1 (4.2–6.4)	<0.01
**BUN, median (IQR), mg/dL**	14 (11–16)	15 (13–17)	13 (11–16)	<0.01
**Creatinine, median (IQR), mg/dL**	0.8 (0.7–1)	0.9 (0.8–1.1)	0.8 (0.7–1)	<0.01
**TGL, median (IQR), mg/dL**	97 (68–140)	109 (85–143)	90 (63–139)	0.029
**Chol, mean ± SD, mg/dL**	204.22 ± 33.47	205.10 ± 34.71	203.87 ± 33.06	0.780
**LDL, mean ± SD, mg/dL**	136.44 ± 34.19	137.1 ± 34.54	136.19 ± 34.13	0.84
**HDL, median (IQR), mg/dL**	45 (38–58)	44 (35–55)	46 (39–58)	0.091
**HBA1c, median (IQR), %**	5.5 (5.3–5.8)	5.6 (5.4–5.9)	5.5 (5.3–5.7)	0.038

SD, standard deviation; BMI, body-mass index; SBP, systolic blood pressure; DBP, diastolic blood pressure; bpm, beats per minute; FBS, fasting blood sugar; URCA, uric acid; BUN, blood urea nitrogen; TGL, triglyceride; Chol, cholesterol; LDL, low-density lipoprotein; HDL, high-density lipoprotein; CRP, C-reactive protein; HBA1c, glycated hemoglobin; RI, resistance index; ECA, external carotid artery; ICA, internal carotid artery; IQR, interquartile range; CCA, common carotid artery; VA, vertebral artery; CP, carotid plaque; cPS, carotid plaque score; IMT, intima media thickness; *p*, *p* value for Fisher’s Exact test, ANOVA or Mann-Whitney U tests.

**Table 2:** Pearson correlation coefficients between the triglyceride glucose (TyG) index and parameters of carotid ultrasound in community-dwelling individuals.

Pearson Correlation Coefficients, *N* = 276 Prob > |*r*| under H0: Rho = 0					
	cPS	CCA IMT	ICA IMT	ECA IMT	TyG
**TyG**	0.15	0.27	0.20	0.22	1
***p* Value**	0.01	<0.01	<0.01	<0.01	-

cPS, carotid plaque score; CCA, common carotid artery; ICA, internal carotid artery; ECA, external carotid artery; IMT, intima media thickness.

**Table 3:** Odds ratio (OR) of multiple logistic regressions for high internal (ICA), external (ECA), and common (CCA) carotid artery intima media thicknesses (IMTs) (cm) and the presence of carotid plaque in community-dwelling individuals.

CCA IMT > 0.61					ICA IMT > 0.44				ECA IMT > 0.38				Plaque Presence			
Variable	OR	95% CI		*p*	OR	95% CI		*p*	OR	95% CI		*p*	OR	95% CI		*p*
**Age**	1.06	1.02	1.09	<0.01	1.02	0.99	1.05	0.127	1.00	0.97	1.03	0.561	1.14	1.10	1.18	<0.01
**Sex (M)**	3.53	1.82	6.86	<0.01	5.39	2.99	9.74	<0.01	3.24	1.73	6.07	<0.01	1.83	0.99	3.37	0.051
**BMI**	1.00	0.89	1.12	0.968	1.09	0.98	1.21	0.104	1.07	0.95	1.19	0.224	1.36	0.75	2.46	0.119
**TyG**	2.09	1.07	4.09	0.029	0.93	0.52	1.65	0.808	1.01	0.55	1.84	0.973	0.92	0.82	1.02	0.296
**HTN**	2.48	1.24	4.93	<0.01	1.21	0.62	2.36	0.559	1.65	0.83	3.28	0.151	2.36	1.15	4.85	0.018

TGL, triglyceride; Chol, cholesterol; LDL, low-density lipoprotein, HDL, high-density lipoprotein; HTN, hypertension; TyG, triglyceride glucose index; M, male; *p*, *p* value, OR, Odd Ratio; CI, Confidence Interval.
